# Optimized protocol for live imaging of *Bacillus subtilis* phage infection at a single-cell resolution

**DOI:** 10.1016/j.xpro.2026.104805

**Published:** 2026-08-26

**Authors:** Hannah R. Bonham, Virginie Grosboillot, Valentina A. Floccari, Iztok Dogša, Anna Dragoš

**Affiliations:** 1 Department of Microbiology, Biotechnical Faculty, University of Ljubljana, 1000 Ljubljana, Slovenia

**Keywords:** Microbiology, Microscopy, Model organisms

## Abstract

Time-lapse microscopy of growing bacteria allows quantitative observation of cellular features and dynamic processes. Here, we present a protocol for live imaging of *Bacillus subtilis* phage infection at single-cell resolution. We describe steps for preparing agarose-based solid media for microscopy, staining phage particles with a DNA-binding dye, and making agarose pads for single-cell growth analysis. This protocol is optimized for studying growth and infection of the oxygen-dependent bacterium *Bacillus subtilis* using the lytic phage Nf.

## Highlights

• Guidance on time-series imaging of phage infection dynamics at single-cell resolution
• Steps for live imaging of *Bacillus subtilis* phage infection at single-cell resolution
• Agarose pads preparation, fluorescent-labeled phage, and bacteria-phage co-culture
• Image-postprocessing for background fluorescence and spectral cross-talk correction

## Abstract (editor highlights)

Publisher’s note: Undertaking any experimental protocol requires adherence to local institutional guidelines for laboratory safety and ethics.

## Graphical abstract

**Figure:**
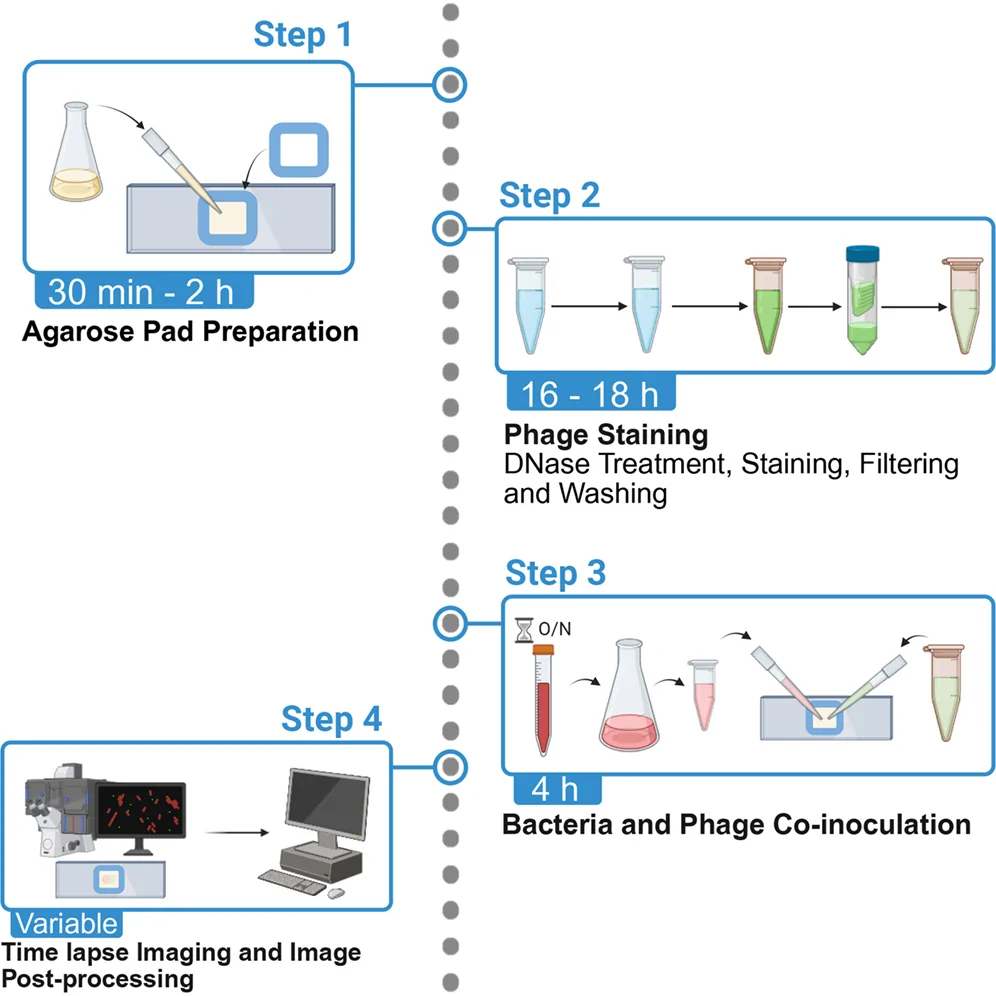

## Before you begin

There are a number of requirements needed to capture bacterial cell division and death events under the microscope, such as: 1) an appropriate initial physiological state of the cells; 2) the correct concentration of cells; 3) maintenance of sample stability for consistent microscopy focus; and 4) adequate oxygen and humidity levels.

In the past decade microscope technology has advanced to the point where high spatial resolution in combination with long temporal coverage can be achieved. Despite this progress, applying this technology to live-cell imaging on solid media specifically remains challenging. Fixation or physical immobilization of cells on a solid surface inhibits growth, while cell dispersal in liquid culture makes it difficult to maintain focus on one cell or group of cells.

This protocol contains several subsections, including: 1) agarose pad preparation using adhesive frames (Thermo Scientific™ Gene Frame)^1^; 2) staining of phage particles; 3) bacteria and phage inoculation; and 4) post-processing. These methods are optimized for observing phage–host interactions at the single-cell level using fluorescent microscopy techniques, enabling tracking of both phage virions and bacteria on solid media without fixation or physical immobilization.

To improve accessibility for readers with colour vision deficiencies, fluorescence acquired in the red channel (561 nm), is displayed in magenta, and phage fluorescence acquired in the green channel (488 nm), is displayed in cyan.

### Innovation

Until now, phage–bacteria interactions have largely been studied using indirect, plating-based, population-level assays, more recently complemented by endpoint imaging approaches. Here, we present a set of optimized protocols for real-time investigation of phage infection dynamics. The advance of our study lies in shifting from traditional bulk measurements toward imaging-based, single-cell resolution analysis of phage infection. This enables a more detailed and quantitative comparison of infection parameters, opening new possibilities for studying phage–host interactions. While further quantitative development is still needed, these protocols set a foundation for future analyses for other phage–host systems and for adapting the workflow to higher-throughput experiments.

## Key resources table

**Table:** 

REAGENT or RESOURCE	SOURCE	IDENTIFIER
**Chemicals, peptides, and recombinant proteins**		

LB Broth	condalab	Cat#1231.00
Agarose	Sigma Aldrich	Cat#A9539-250G
Sodium phosphate dibasic (Na_2_HPO_4_)	Sigma Aldrich	Cat#S5136-500G
Potassium dihydrogen phosphate (KH_2_PO_4_)	Supelco	Cat#1.04873.1000
Sodium chloride (NaCl)	Honeywell	Cat#31434_1KG
Ammonium chloride (NH_4_Cl)	Sigma Aldrich	Cat#A4418-500G
Trizma® hydrochloride (Tris-HCl)	Sigma Aldrich	Cat#102560344
Magnesium sulfate heptahydrate (MgSO_4_)	Supelco	Cat#1.05886.1000
FM™ 4-64 Dye (N-(3-Triethylammoniumpropyl)-4-(6-(4-(Diethylamino) Phenyl) Hexatrienyl) Pyridinium Dibromide)	ThermoFisher Scientific	Cat#T13320
DNase I, RNase-free (1 U/μL)	ThermoFisher Scientific	Cat#EN0521
SYBR™ Gold Nucleic Acid Gel Stain	ThermoFisher Scientific	Cat#S11494

**Other**		

Glass Microscope Slides	VWR	Cat#HECH91240000252
Thermo Scientific™ Gene Frame, 65 μL (1.5×1.6 cm)	ThermoFisher Scientific	Cat#AB0576
Coverslips for sticky-Slides (Polymer)	ibidi	Cat#10813
SafeSeal reaction tube, 1.5 mL, PP	Sarstedt	Cat#72.706.001
ZEISS Elyra 7 super-resolution microscope, α Plan-Apochromat 63×/1.46 Oil Korr M27 objective, Edge 4.2 SIM camera	Carl Zeiss Microscopy GmbH	Model: Elyra 7
Amicon® Ultra Centrifugal Filter, 100 kDa MWCO	Sigma Aldrich	Cat#UFC9100

**Bacterial and virus strains**		

Species: *Bacillus subtilis;* Strain: P9_B1 amyE::mKate (spec)	Dragoš et al^2^	N/A
Strain: *Bacillus* phage Nf	Bacillus Genetic Stock Center	N/A

**Software and algorithms**		

Fiji ImageJ, version 1.54p	Schindelin et al^3^	https://imagej.nih.gov/ij/
ZEN lite software, version 3.13.109.02000	Carl Zeiss Microscopy GmbH	N/A

## Materials and equipment

**Table:** Preparation of 5x M9 Salts in 100 mL

Reagent	Final concentration	Amount
Sodium phosphate dibasic (Na_2_HPO_4_)	211 mM	3.0 g
Potassium dihydrogen phosphate (KH_2_PO_4_)	110 mM	1.5 g
Sodium chloride (NaCl)	42.8 mM	0.25 g
Ammonium chloride (NH_4_Cl)	93.5 mM	0.5 g
dH_2_O	NA	Up 10 100 mL
Total	NA	100 mL

***Note:*** Store at –4C or room temperature for up to 3 months.

**CRITICAL:** Ammonium chloride (NH_4_Cl) is a hazardous chemical, acute toxic (category 4, harmful if swallowed) and is an eye irritant (category 2, cause serious eye irritation). When working with this chemical, wear protective equipment to prevent ingestion (N95 mask) and protective eye wear in addition to normal protective wear (gloves and lab coat).

**Table:** Preparation of SM buffer in 500 mL

Reagent	Final concentration	Amount
Sodium chloride (NaCl)	100 mM	2.92 g
Trizma hydrochloride (Tris-HCl)	50 mM	3.94 g
Magnesium sulfate heptahydrate (MgSO_4_)	8.0 mM	0.98 g
dH_2_O	NA	Up to 500 mL
Total	NA	500 mL

***Note:*** Store at –4C or room temperature for up to 6 months.

**Preparation:** Place a up to 300 mL of ddH_2_O in a beaker before adding the reagents. Using mechanical or magnetized stirrers (recommended) to dissolve the reagents before being the total volume up to 500 mL. After, adjust the pH of the solution to 7.5, then filter using 0.22 μm filters into a sterilized container.

**CRITICAL:** None of the reagents have a reported hazard, when handling use standard safety protection including to normal protective wear (gloves and lab coat), protective eye wear and standard face mask.

## Step-by-step method details

### Preparation 1: Agarose pad preparation using an adhesive frame

**Timing: Approximately 30 min to 2 h, depending how the medium is sterilized.**

In preparation 1, we describe how to prepare the agarose pads using the adhesive frames. We also describe two possible media for the pads, the addition of dyes and a method for storing the pads.

**Figure 1:**
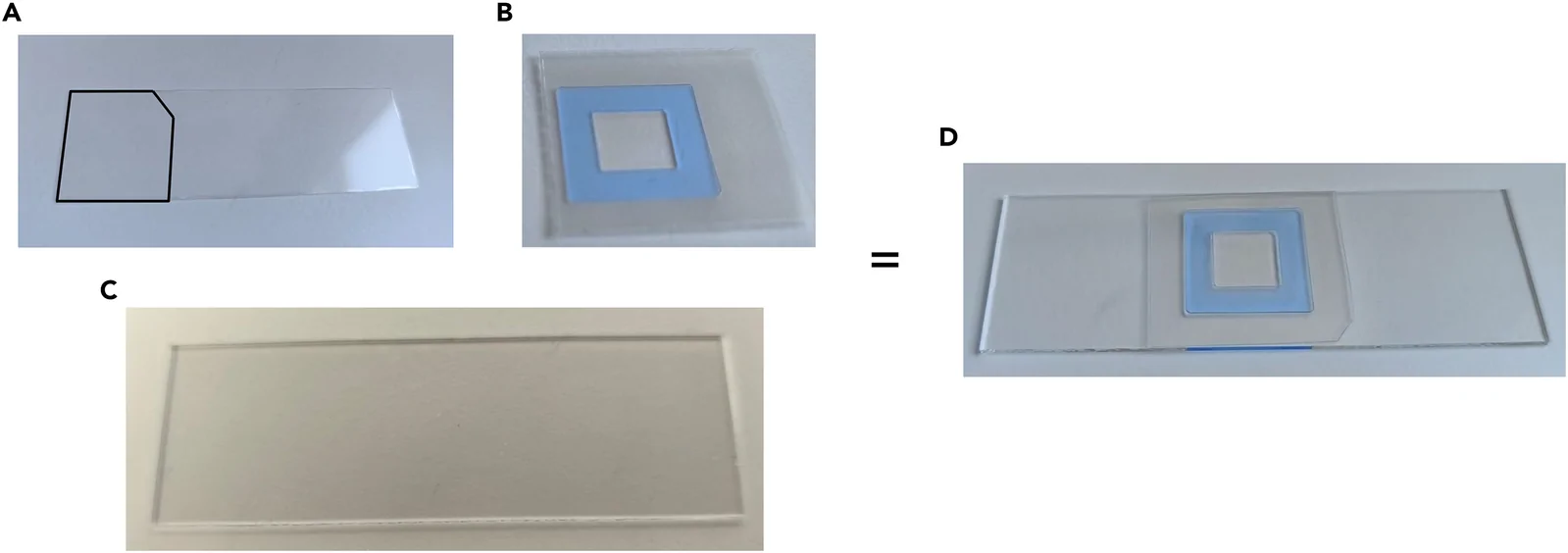
Agarose Pad Preparation Equipment used to assemble the agarose pad on the microscope slide. (A) ibidi Coverslips for sticky-Slides (Polymer); gas-permeable, flexible and can be cut to size (black square). (B) Adhesive frames (blue square) covered with plastic foil on both sides to protect the adhesive material. (C) Generic glass microscope slide, 76×26 mm. (D) The final assembled microscope slide (including a 1.5% agarose medium pad) ready for the microscope. See also Methods Video S1.
  a. Thoroughly clean the microscopy glass slide using 70% ethanol and paper towel.
    ***Note:*** Different chemical cleaning methods (i.e. KOH based cleaning) can be used, the main goal is to remove any oil or fat residues from manufacturing or handling. Also to keep the slide sterile while preparing the agarose slides.
    ***Note:*** The frames are adhesive, which enables strong adhesion to the glass slide and coverslip. They are stored between two sheets of clear plastic. Remove the plastic side that **does not** contain a hole in the center, maintaining the other plastic sheet in place. This can be made easy by folding the sheet edge down completely and roll it away from the pad. The frames are 16×15 mm with a thickness of 0.25 mm and hold a volume of 65 μL.
  b. **After removing the plastic cover** (lacking the square opening) place the adhesive side of the frame in the middle of the glass slide. Press down on the frame (covered by plastic sheet) to confirm it is properly sealed to the glass slide. Do not remove the second plastic cover sheet.
  Methods Video S1. Building the slides Instructional video on each step of the Agarose pad preparation using adhesive frame, including storage and sample inoculation. Video is for demonstration purposes in unsterile conditions.
3. Filling the adhesive frames with the agarose medium.
  a. Pre-heat an Eppendorf tube to 60 °C using thermoblock.
    ***Note:*** 10 min in most cases is sufficient.
  b. Fill the preheated Eppendorf tube with 150 μL of the agarose medium. Altought only 100 μL is required. Preparing 1.5x volume reduces the risk of bubbles when mixing reagents into the 1.5% agarose medium and pipetting.
  c. While keeping the Eppendorf tube containing the medium in the thermoblock, carefully pipette 100 μL of the medium into the middle of the adhesive frames.
    ***Note:*** Leave a small amount of the medium in the tip to prevent bubbles from forming in the medium.
  d. Immediately place a sterile empty glass slide on top of the pad.
    ***Note:*** The liquid media will spread over the entire adhesive frames and onto the glass slide, this is expected.
    **CRITICAL:** This step needs to be done quickly, if the agarose media begins to set before placing the glass slide, the cover slip cannot be added without squishing the pad and sample. Steps to increasing the room temperature or incubating the glass slides (30 °C) before adding the agarose media can assist if the media is setting too quickly.
  e. Leave the agarose pad to set for 20 min.
    ***Note:*** For low melting agarose, 5 to 10 min is a sufficient amount of time to let it set.
    **CRITICAL:** If using UV sensitive reagents, keep the samples in the dark as much as possible. Preparing the glass slide should be performed in sterile conditions or as sterile as possible.
4. Microscope slide storage.
  a. Carefully pick up the two glass slides (the top weight slide and slide with the frame and pad), keeping them together to prevent the two glass slides from coming apart. Place the slides on aluminum foil and carefully wrap the slides. If the glass slides remain secure and tightly wrapped in aluminum foil it will keep the agarose pad protected and prevent the pad from drying, allowing long-term storage.

***Note:*** LB and M9 agarose pads can be stored at 4°C for up to 8 weeks in the right conditions. It is recommended to perform your own tests to determine what length of storage time is acceptable for your experiments and environment.

***Note:*** If using a different medium or medium containing dyes, it is recommended to test how long the medium can be stored.

***Note:*** Microscope slides are stored with the second glass slide placed on top, sealing the pads and preventing them from drying out and from contamination. As stated above, the slide can be stored in these conditions for a prolonged period of time (8 weeks). However, be aware that the pads will eventually dry out and shrink over time. The second glass slide is removed before adding the biological samples.

1. Preparation of solid growth medium for agarose pad or multi-pad slides.
  a. For LB-1.5% agarose: Add 1.5 g of agarose powder to 100 mL of LB medium. Autoclave. Store at 60°C to prevent the medium from solidifying.
    ***Note:*** Recommended to prepare pads immediately after sterilizing the media. Medium should not be stored for more than 2 days.
  b. For M9-1.5% agarose: Add 1.5 g of agarose powder to 100 mL of sterile M9 medium. Microwave to melt the agarose. Store at 60 °C to prevent the medium from solidifying.
    i. M9 medium (100 mL volume) preparation: Into 77.79 mL of sterile MilliQ water add 20 mL of sterile M9 salts (Detailed in materials and equipment), 200 μl of sterile 1M MgSO_4_, 2 mL of sterile 20% glucose, 10 μL of sterile 1M CaCl_2_.
      ***Note:*** The media can be readily interchanged. Consider the requirements needed to prevent contamination and reduce background fluorescence. Protocols were optimized using LB and M9 minimal media. LB medium results in higher background fluorescence, especially in the GFP fluorescence range compared to M9 minimal medium. However, a limitation with M9 minimal medium is a high risk of contamination, due to multiple preparation steps. Therefore, it is highly recommended to use fresh M9-agarose pads or wells. Agarose is recommended due to it purity and finer texture compared to agar, which allows to obtain smoother and more optically uniform surface. The agarose powder used in this preparation had a gelling temperature of 36 °C.
2. Positioning the adhesive frame on the glass slide (Figure 1).

**CRITICAL:** The adhesive frames (Thermo Scientific™ Gene Frame, 65 μL (1.5×1.6 cm)) used in this protocol were purchased from ThermoFisher Scientific (AB0576). However, as of late 2025 this product has been discontinued, alternative products can be purchased from different manufactures, for instance: SecureSeal™ Frame and Cover from GRACE BIO-LABS and iSpacer frames from SUNJin Lab Optical Clearing Innovation.

5. Preparing Agarose Pads that contain FM 4–64 Dye.
  ***Note:*** Certain dyes can be added to the agarose pad for cell visualization. This protocol has been optimized for using FM™ 4–64 Dye (Thermofisher, Catalog number T13320) in the agarose pads. FM 4–64 Dye binds to the phospholipid bilayer in bacterial cell membrane, producing red fluorescence (excitation ∼515–530 nm/ emission ∼640–660 nm,) allowing to visualize cell membrane.
  a. After taking the aliquot of agarose media and placing it in the thermoblock (see Preparation 1. 3d. add FM 4–64 to a final concentration of 1 μg/μL. Proceed to next steps of the protocol.
    **CRITICAL:** While the pad is drying, cover it with aluminum foil to protect the FM 4–64 stain from the light. Pads prepared with additional reagents (dyes) can reduce the possible storage time. When adding light sensitive reagents to the pads, keep them covered as much as possible to protect the pads from the light.

### Preparation 2: Staining of phage particles

**Timing: 18 h, including 12 h overnight incubation.**

Here, we describe the protocol for staining phage particles with fluorescent dyes. Including steps to remove host DNA contaminant from the phage sample and excess unbound dye.

***Note:*** This protocol was optimized for staining of *Bacillus* phage Nf using SYBR™ Gold Nucleic Acid Gel Stain.

6. DNase I Treatment. Troubleshooting 2.
  a. Place 1 mL of phage lysate in a light protective Eppendorf tube, add 111 μL of 10x DNase I buffer (final conc. 1x) and 20 μL of DNase I (1 U/μL). Mix sample gently by inverting or pipetting up and down.
    ***Note:*** Phage lysate concentration used during development of this assay was 9×10^8^ PFU/mL.
  b. Incubate at 37 °C for 1 h.
    ***Note:*** This step removes an excess of free genomic DNA often following bacterial lysis. If performing for the first time, include controls to confirm phage viability after DNase treatment. If the stain being used does not bind to DNA, this step can be skipped.
    ***Note:*** These protocols do not include a DNase I inactivation step since it was confirmed that the DNase I remaining in the same did not inhibit dye binding or phage function.
7. SYBR gold phage staining and washing. Troubleshooting 2.
  a. After DNase I treatment, add 1.2 μL of SYBR gold nucleic acid gel stain from 1,000x working stock (final concentration 1x) to the DNase treated phage lysate sample. Invert sample 5 times then incubate for 14–16h at 4°C.
  b. The next day remove the stained phage sample from 4°C and place it on ice. Also place the centrifugal filter (one per sample) and the SM buffer on ice.
    i. Transfer the stained phage sample to the upper reservoir of the ultra centrifugal filter, add 4 mL of SM buffer to the upper reservoir.
      ***Note:*** The centrifugal filter used in these experiments was the Amicon® Ultra Centrifugal Filter.
    ii. Centrifuge the sample for 3200 *g* for 1 min at 4°C.
      **CRITICAL:** Do not let the volume of the sample in the upper reservoir fall below 1.5 mL. If all the solution pass through the filter, the filter will dry and ruin the sample. Monitor the volume in the upper reservoir during the centrifugation steps.
    iii. After centrifugation, add 4 mL of fresh SM buffer to the upper reservoir and repeat the centrifugation step 15 more times.
      ***Note:*** The number of washes was optimized for removing as much unbound SYBR Gold stain as possible from the sample to minimize background fluorescence. Different stains could require less wash steps.
    iv. After completing the wash steps, centrifuge the sample until its volume is reduced to 1 mL. Harvest the sample by pipetting the sample 5 times along the filter walls to dislodge any stained phage particles which could have attached to the filter.
    v. Transfer the sample to 1.5 mL light protective Eppendorf tube and store at 4°C.
      **CRITICAL:** It is highly recommended to perform additional phage activity test step (plaque assay) to confirm the phage infectious titer after treatment, staining and filtration. The phage Nf, used in the development of this protocol, is not affected by steps of the staining, however, this may not be true for other species of phage. Troubleshooting 3.

### Preparation 3: Microscope slide preparation with bacteria and phage co-inoculation onto agarose pad

**Timing: Approximately 5–6 h.**

In preparation 3, we describe the culture conditions used for Bacillus subtilis growth for time-lapse imaging. The specified culture dilution provides an optimal starting concentration for time-lapse imaging of bacteria growth. These steps also include a guide for applying cell culture and phage samples onto agarose pads.

***Note:*** To improve imaging results, it is recommended to use a combination of stains and/or fluorescent reporters for which the emission wavelength spectra does not overlap. All experimental protocols were optimized using bacteria constitutively expressing *mKate* gene and lytic phages stained with SYBR Gold.

8. Day 1: Preparation of *Bacillus subtilis* starter culture.
  a. Inoculate the culture directly from −80°C glycerol stock into 5 mL of LB culture tube.
    ***Note:*** Inoculating directly from the glycerol is performed due to the very fast mutation rate of *Bacillus subtilis* used during the development of these protocols. For less mutation prone bacterial species, it is recommended to streak onto a plate first and select a single colony to culture.
  b. Incubate for up to 14–16 h with shaking at 220 rpm at 37°C.
9. Day 2: Re-cultivation to mid-exponential growth phase and transfer onto agarose pad.
  a. Re-inoculate culture into fresh medium using dilution factor of 1:100.
  b. Incubate with shaking at 220 rpm at 37°C. The culture will take approximately 2h to 2:30 h to reach OD_600_ = 0.4.
    **CRITICAL:** Aim for OD_600_ between OD_600_ = 0.4 to OD_600_ = 0.6.
  c. Minimum 2 h before the culture reaches the right OD_600_, remove the stored microscopy slide (see Preparation 1) from 4°C and incubate it at the temperature at which you are planning to run your time-lapse growth experiment.
    **CRITICAL:** Leave the slide wrapped in aluminum foil.
    ***Note:*** It is best to remove the stored microscope slide from 4°C and incubate it at the desired temperature before moving on with any further steps on Day 2. This is to prevent condensation forming if placed in a temperature-controlled microscope and to maintain cell health when adding the sample to the pad.
  d. After the culture has reached OD_600_ ≈ 0.4, dilute the culture to OD_600_ = 0.15.
    ***Note:*** OD_600_ = 0.15 was determined to be the optimal culture density to initiate time-lapse cell imaging of phage infection.
  e. Prepare a new 1.5 mL Eppendorf tube with 1 mL of sterile LB media and preheat at 37 °C. The calculation for diluting the culture to OD_600_ = 0.15 is:
    Bacteria Concentration for Phage Infection (Final OD_600_ = 0.15):
    $$\frac{OD600\;culture}{0.15}=dilution\;factor\to \frac{1000\;\mu L\;}{dilution\;factor}=x\;\mu L$$
  f. After preparing the diluted culture (OD_600_ = 0.15), remove the microscope slide from the aluminum foil and gently slide the top glass slide off the agarose pad.
    **CRITICAL:** Perform the following steps in sterile conditions to prevent contamination of the pad. Also, if using UV sensitive reagents, keep the samples in the dark as much as possible.
    ***Note:*** Move the glass slide off horizontally rather than directly upwards off the pad. This will help prevent the pad from sticking to the glass cover and keeping it in the adhesive frame.
  g. Using a paper towel remove any excess agarose from the plastic cover of the adhesive frames and glass slide. Peel-off the plastic cover from the adhesive frames - this will expose the adhesive on the top side of the frame.
    ***Note:*** Sometimes the 1.5% agarose pad will stick to the plastic cover. A pipette tip can be used to separate the pad from the plastic cover.
  h. Place the slide in a sterile location and allow it to further dry for a period of 7–10 min.
    **CRITICAL:** It is strongly recommended to use this drying step for minimum 7 min. This will remove any additional moisture left on the pad that could cause moisture to build up after the cover slip is added.
  i. Spot 0.5 μL stained phage sample on one side of the pad. Leave the sample to dry for 2 min.
  j. On the second half of the pad, place 0.5 μL of bacteria, allowing the two samples to overlap slightly in the middle of the pad. Leave the sample to dry for 2 min or longer, until fully dry.
    ***Note:*** For these experiments, the phage suspension was diluted to produce a 1:1 volume ratio with the bacteria when plated. Depending on the type of phage (lytic vs temperate, high virulence level, etc.), MOI optimization should be performed.
  k. Remove the protective foil from the coverslip and place it (protected side face down) on top of the adhesive frame, lightly/firmly pressing around to seal the frame to the cover slip. Troubleshooting 1.
    ***Note:*** Use your finger to gently press around the far edges of the adhesive frame (not directly on the agarose pad), this will fully seal the adhesive frame to the cover slip.
    **CRITICAL:** When placing the cover slip, start by lowering one side then lower the other like a phone screen protector. It is important that there are no air gaps between the coverslip and the pad.
10. The microscope slide is ready for observation. Troubleshooting 5.

The fluorescent microscope images displayed in Figure 3 represent frames of a time lapse experiment performed using a *Bacillus subtilis* mKate expression strain (magenta) combined with SYBR™ Gold stained Nf phage (cyan) (see also Figures S1 and S2). Images where acquired using a ZEISS Elyra 7, the microscope was operated in wide-field mode. The green and red fluorescence was detected using 488 nm diode laser 100 mW combined with 495–550 nm bandpass emission filter and 561 nm diode laser 100 mW combined with 570–620 nm bandpass filter with an additional long-pass 655 nm filter, respectively, applying 7% laser power for both lasers. Images were acquired using a Plan-Apochromat 63×/1.4NA oil DIC M27 objective. Images were recorded using two monochrome cameras operating in integration mode with **50 ms exposure time** per channel, **1×1 binning**, **16-bit** digitization, and **1280×1280 pixel** image size. Camera digital gain was set to **1×**, amplifier gain to **0**, amplifier offset to **0.1**, and digital offset to **0**. The acquisition used a pixel size of **96.8 nm×96.8 nm** in the xy plane (field of view approximately **124×124 μm**), with no digital zoom (1×). Images were collected as a time series of **320 frames** at **3-minute intervals**, corresponding to approximately **16 h** of continuous imaging. Six stage positions were imaged under identical settings. The incubation chamber was set to 32°C during the time lapse experiment.

### Preparation 4: Image post-processing

**Timing: Processing time is dependent on quantity of image data (exact length of the experiment and number of time series).**

Here, we describe the final steps of the protocol, involving image processing to remove background fluorescence and fluorescence cross-talk between channels. We explain the control images required to determine a baseline for each signal independently. These corrections improve the accuracy of fluorescence measurements and enhance image clarity.

**CRITICAL:** All image data were saved in original ∗.czi format (Zeiss proprietary format) and further processed using Fiji ImageJ v. 1.54p 17 February 2025,^2^ Fiji. All files described in this protocol are available at Zenodo: https://zenodo.org/records/18165781.

***Note:*** The controls include individual samples of both the SYBR Gold-stained phage particles and *B. subtilis* expressing mKate. Both individual samples are imaged with red and green channels. Control images should be acquired with same microscopy settings as the time lapse sample using both fluorescence channels.

***Note:*** The autofluorescence of the samples themselves should also be taken into consideration. Bacteria and phage likely contain features that naturally fluoresce at certain wavelengths and can increase over time. But because of the phage sample’s weak signal it was decided to forgo further post processing, specifically the autofluorescence.

11. Control Images – single sample only.

Bacteria_only – sample of *B. subtilis* mKate, without phages; imaged using the green (**Bacteria_only_Green**) and red channel (**Bacteria_only_Red**).

Phages_only - sample of stained Nf phage, without bacteria; imaged using the green (**Phages_only_Green**) and red channel (**Phages_only_Red**).

***Note:*** Before post-processing, time-lapse images were subjected to flat-field corrections, which in this case only produced a small improvement in image quality. Flat-field correction can be performed using previously described fluorescence microscopy correction methods.^4^ Because flat-field corrections are a standard image correction step, it is not included in the details for these methods.

12. Determine average background fluorescence for each channel.
  ***Note:*** Background fluorescence is the observed baseline fluorescence, which is not generated by a fluorescent object due to fluorescent label or dye. It can be generated by camera noise, autofluorescence of the sample/agarose/media or out-of-focus light. To isolate true fluorescence signal originating from fluorescent label or dye, background fluorescence noise needs to be removed. The background fluorescence value is calculated from control samples, **Bacteria_only_Red** and **Phage_only_Green**.
  ***Note:*** Background fluorescence can vary over time, but our aim is to reduce it to a low signal (20) not to 0 completely.
  a. Open both control images in Fiji ImageJ, **Bacteria_only_Red** and **Phage_only_Green.**
  b. Go to Analyze → Tools → ROI Manager.
  c. Use the Rectangle tool to draw 5 large Region Of Interests (ROIs) in background areas on an image, without fluorescent objects.
    **CRITICAL:** Add each ROI to the ROI Manager. Keep ROI size consistent and save. Avoid image edges and uneven illumination, especially if no flat field correction has been performed.
  d. With all 5 ROIs selected, go to Analyze → Measure (shortcut: M).
  e. Record the mean intensity for each ROI, average the ROI’s mean intensities for each channel in order to obtain average green and red channels background values. These mean values now act as your BG_G and BG_R values for the background fluorescence for both channels.
    BG_G = average green background fluorescence value taken from **Phage_only_Green** image measurements (average from 5 or more measurements).
    BG_R = average red background fluorescence value taken from **Bacteria_only_Red** image measurements (average from 5 or more measurements).
13. Remove background fluorescence from recorded time series.
  a. **Open the recorded time lapse** in Fiji.
    **CRITICAL:** Depending on the software being used, Fiji will open the time lapse file (.czi) into two stacks, for each channel (red and green). The stacks should also include a slider for scrolling through each frame. All measurements are performed on the first image in the time lapse, assuming the background is constant through time. If Fiji opens it as a hyperstack, red and green channel in one window. Convert to 32-bit image then uses Image → Colour → Split Channels to separate the stacks.
  b. Convert both stacks to **32-bit images**: Image → Type → 32-bit.
  c. Save and make duplicates of both channels: e.g., **date_p#_32bit_green_channel**.
    ***Note:*** To keep track of image files after completion of post-processing step, keep a record for the acronyms used.
    BS = background subtracted.
    CTS = cross talk subtracted.
    G/R_stack= which channel, green channel or red channel.
  d. Remove the background fluorescence from both channels (see Preparation 5.1):

Green channel: Process → Math → Subtract… → value BG_G “Process all slices.” → save as G_stack_BS_date_p#_sample.

Red channel: Process → Math → Subtract… → value BG_R → “Process all slices.” → save R_stack_BS_date_p#_sample.

**CRITICAL:** In most cases, the background intensity is ideally reduced to 0. However, because of the stained phage signal is very weak in this post-processing we aim to keep the background signal at a range of 15–25 to avoid over-subtraction and loss of the weak phage signal.

***Note:*** Files generated in Additional protocol 2.d (directly above) are the green/red channel stacks that have only had the background removed, the green channel still contains cross-talk signal. After removing the background from the red channel, it was determine that it did require additional signal subtraction as the cross-talk for red channel is minimal/not visible (strained phage signal appearing in the red channel).

***Note:*** It is recommended to save copies at each step, especially after modification in case of mistakes during processing.

14. Determine the general constant cross-talk factor (k) and generate an intensity scaled time-series images for removing the majority of the cross-talk.
  The cross-talk is not uniform across the green channel image, it is spatially correlated with mKate fluorescent objects (the bacteria). To remove the cross-talk present in the green channel, the cross-talk factor (k) will be calculated (equation bellow) using fluorescent values at locations where only the cross talk is present on the red and green channel (same location). After calculating k, an intensity scaled red channel images of the time-lapse is created and is used to subtract the signal from the green channel (Figure 4A).
  a. Open the ROI manager and chose 10 spots, including 5 spots on the background and 5 spots on the bacteria on the **G_stack_BS_date_p#_sample** stack. Save the ROI positions.
  b. Using **G_stack_BS_date_p#_sample** (first image), select 10 locations (same size area) using the ROI manager. Five locations on areas without fluorescent objects and five locations overlapping with bacterial cells (avoiding locations with phage present). Aim for areas near the center of the image. Save the ROI locations.
    ***Note:*** For these images it was easier to choose the location on the green channel, since the cross-talk was very visible and the phage particles could easily be avoided.
  c. Open the same ROI locations in the ROI manager on the **R_stack_BS_date_p#_sample** and take the measurements for those locations.
  d. Using the ROI locations on the **G_stack_BS_date_p#_sample** and **R_stack_BS_date_p#_sample** calculate the mean values for the bacteria (fluorescent objects) and background (no fluorescent objects, image background). Perform this for both stacks to obtain G_bacteria and G_background and R_bacteria and R_background:
    **CRITICAL: Reminder!** Because the strained phage signal is so weak, the background signal was kept at a range between 15 - 25.
    G_bacteria = mean of the bacterial signal on the green channel (background subtracted).
    G_background = mean of background signal on the green channel (background subtracted).
    R_bacteria = mean of the bacterial signal on the red channel (background subtracted).
    R_background = mean of background signal on the red channel (background subtracted).
  e. Using these calculated values, the k value can be obtained from the equitation below:
    $$k=\frac{G\_bacteria-G\_background}{R\_\text{bacteria}-R\_background}$$
  f. Generate scaled time-series for experimental cross-talk background removal: Take the **R_stack_BS_date_p#_sample** image: Process → Math → Multiple… → value k → “Process all slices.” → save *R_scale_k,* troubleshooting 4.
  g. Remove cross-talk from green channel: Process → Image Calculator… → Image1: **G_stack_BS_date_p#_sample** → subtract → Image2: *R_scale_k* → “Process all slices.” → save as **G_stack_BS_CTS_date_p#_sample**.
    ***Note:*** You can confirm that the cross-talk has been removed from the green channel by comparing the same signal value locations before and after subtracting the *R_scale_k* image. Using the ROI locations that were used to calculate k (4. d), place them on the green channel after the *R_scale_k* image has been subtracted. Before subtraction the signal on the bacteria would be consistently higher than the background signal (Figure A). After the subtraction, the signal on the bacteria would be almost identical to the background signal value.
  h. After the cross-talk has been successfully removed, the channels (as **G_stack_BS_CTS_date_p#_sample** and **R_stack_BS_date_p#_sample**) can be merged into a hyperstack using Fiji. Additional processing (not included in these instructions) can be performed to include time and scale bars. Time lapses can also be exported as a video file.

## Expected outcomes

Preparation 1: Agarose pads should be fully solidified, smooth and uniform, with solid medium completely covering the square surface within the adhesive frames (Figure 1). No air bubbles, wrinkles and visible dust particles should be present on top of agarose pads, as this can cause the polymer-based cover slip (added over the agarose pad in Preparation 3. to warp.

**Figure 2:**
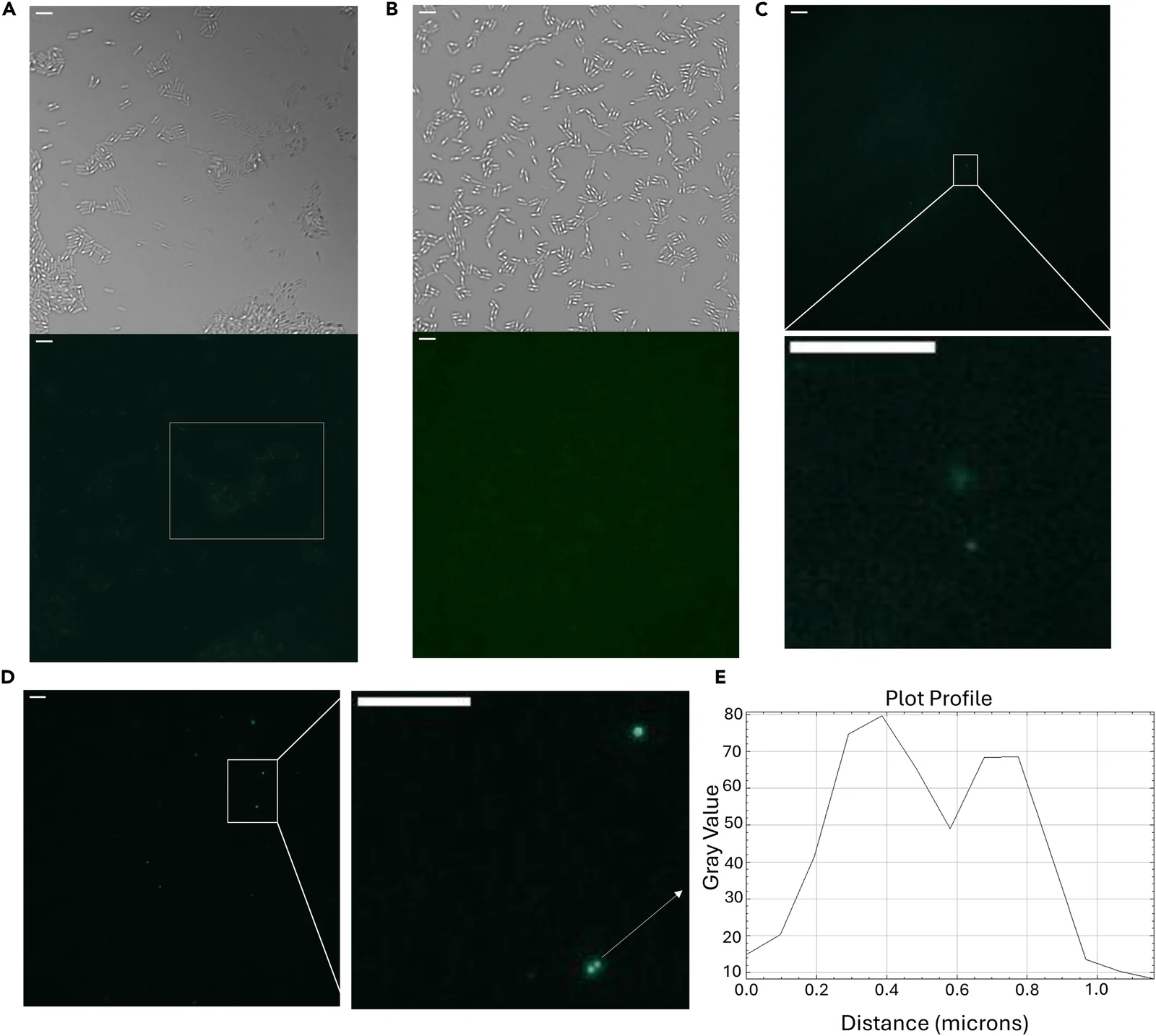
Phage staining by SYBR™ Gold with different treatments and conditions All scale bars (top left corner) correspond to 5 μm. (A) Image of *Bacillus subtilis* cells combined with stain controls sample (phage storage buffer + SYBR Gold), no phage, after 32x volume wash. Top image was taken with bright field (confocal setting) and bottom image with the green channel 488 nm laser. Brightness and contrast were adjusted in Fiji to minimal and maximal displayed values of 50–and 550 (a.u.), respectively. (B) image of *Bacillus subtilis* cells combined with stain controls sample (phage storage buffer + SYBR Gold), no phage, after 60x volume wash. Top image was taken with bright field (confocal setting) and bottom image with the green channel 488 nm laser. Brightness and contrast were adjusted in Fiji to minimal and maximal displayed values of 50–550 (a.u.), respectively. (C) Image of SYBR Gold stained NF phage sample, Including a zoomed in section of the image. Brightness and contrast were adjusted in Fiji to minimal and maximal displayed values 50–1000 (a.u.) (D) Image of SYBR Gold stained NF phage sample, histogram of pixel intensities; x-axis limited to 50–1000 (a.u.), using protocols that included a DNAse treatment including a zoomed in section of the image. Brightness and contrast were adjusted in Fiji to minimal and maximal displayed values 50–1000 (a.u.) (E) Plot profile covering the range of two phage particles present in the Dnase treated sample (Figure 2D) indicted by the white arrow. Plot profile was generated in ZEN lite. See also Methods Video S2.

Preparation 2: This preparation generates a 1 mL sample of SYBR Gold stained phage solution (Figure 2F) that should have a concentration comparable to the phage working stock. Plaque assays should be performed afterwards to quantify functional phage concentration (PFU/mL). During assay development, no loss of infective phages Nf was observed after staining and washing or following DNase treatment. Although fluorescence stability over time has not been formally tested, it was observed that the stained phage samples remain fluorescent for up to 1 week when stored at 4°C and protected from light. However, samples viability under these conditions is specific to NF phage, alternative phage samples may behave differently under the same conditions. For example, a stained phage sample may remain fluorescence for a shorter period of time if the phage is less stable at 4°C.

Methods Video S2. SYBR™ Gold-stained NF phage A Short time lapse video of free floating SYBR™ Gold-stained NF phage after staining, 60x wash step and DNase treatment.

**Figure 3:**
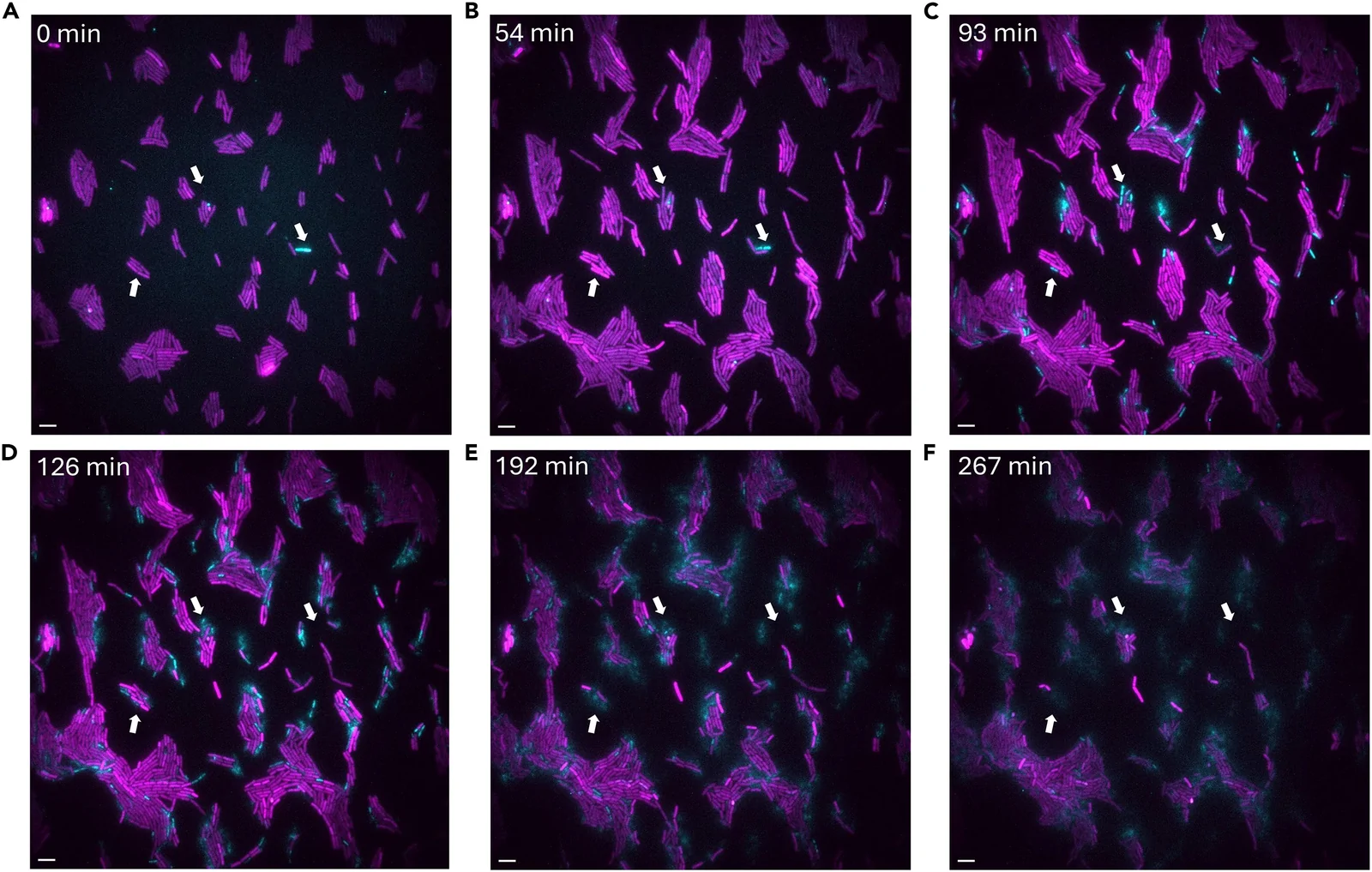
Time-lapse microscopy imaging of *Bacillus subtilis* expressing mKate fluorescence (magenta) combined (1:1) with SYBR Gold stained Nf phage (cyan) All scale bars (bottom left corner) correspond to 5 μm. Combined red (561 excitation wavelength) and green (488 excitation wavelength) fluorescence channel, (A) time point 0 min; (B) time point 18 (54 min); (C) time point 31 (93 min); (D) time point 42 (126 min); (E) time point 64 (192 min); (F) time point 89 (267 min). Images were pseudo colored cyan (SYBR Gold signal) and magenta (mKat**e** signal). Images are described as raw images since they still contain background fluorescent signal and cross-talk. Arrows included in images direct to specific bacterial cells. Single channel images for preprocessed time-lapse image of SYBR Gold stained Nf phage (cyan) and *Bacillus subtilis* expressing mKate fluorescence (magenta) are found in Figures S1 and S2.

Preparation 3: These protocols were developed using a ZEISS Elyra 7 microscope which is equipped with an incubation chamber to maintain the temperature (32 °C) throughout the time-lapse experiment. Combinations of bacteria-phage samples prepared on agarose pads were imaged repeatedly using two fluorescence channels over a period of 16 h. The image acquisition parameters should be adjusted as needed to balance signal intensity, photobleaching, phototoxicity, and temporal resolution. Depending on the manufacturer and model of the microscope being used, additional modifications outside the scope of these methods should be considered. The time-lapse generated should consist of one file containing an overlap of two sets of fluorescent images (red and green channels) (Figures 3A–3F). The equipment used in developing these protocols generated raw ∗.czi microscope files, for analysis and post processing a third-party software (ImageJ) was used. Therefor these instructions are for specifically the third-party software (ImageJ) used, modification to the instructions would be needed if you are using different software.

**Figure 4:**
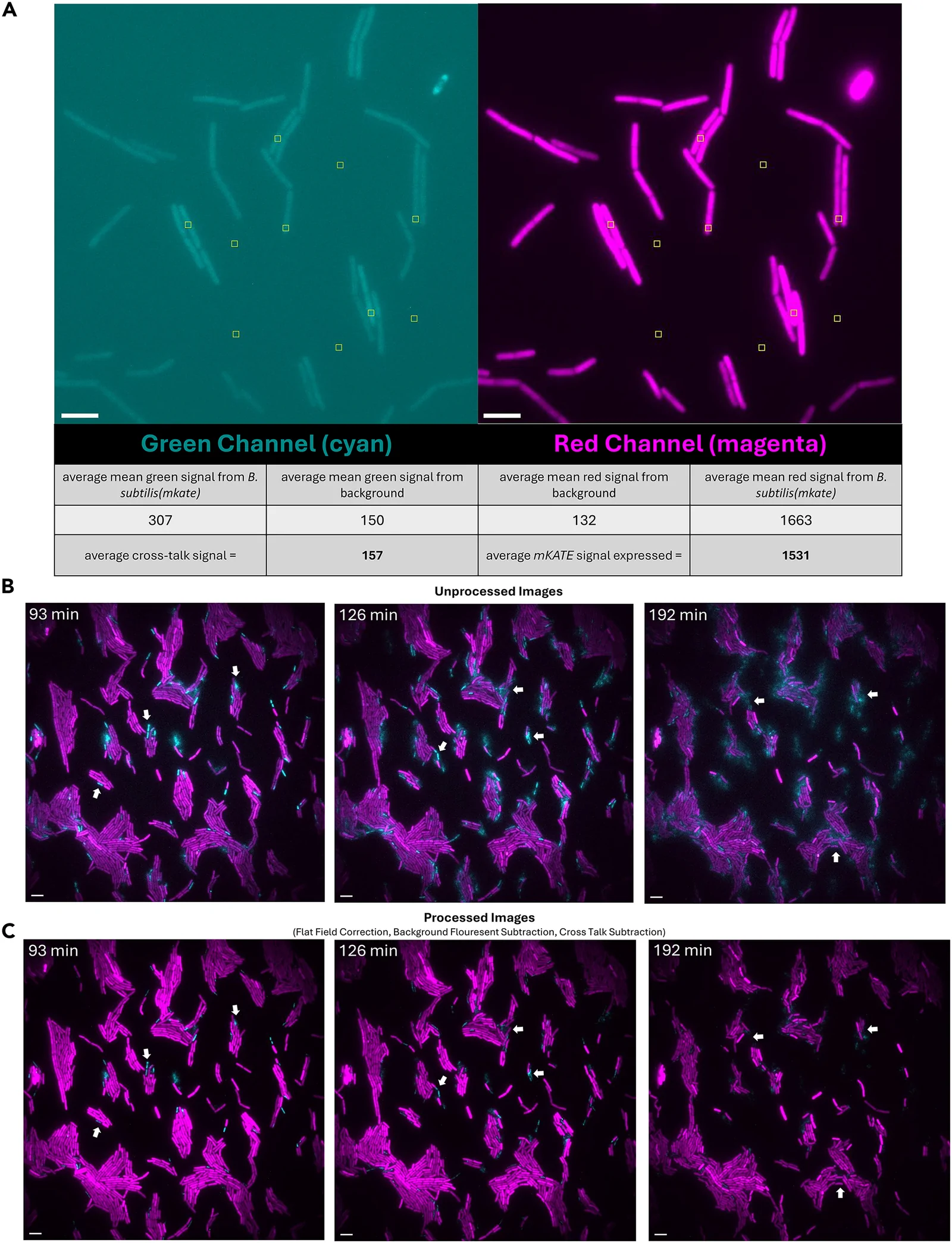
Time-lapse image of *Bacillus subtilis* expressing mKate fluorescence (magenta) combined (1:1) with SYBR Gold stained Nf phage (cyan), before and after post processing All scale bars (bottom left corner) correspond to 5 μm. (A) Control image, green channel image taken with *Bacillus subtilis* expressing mKate fluorescence (no phage), demonstrating an average cross-talk signal of *Bacillus subtilis* (mKate) in the green channel. (B) Unprocessed time lapse images at specific timepoints, 93 min (time point 31), 126 min (time point 126); 192 min (time point 64), with combined red and green channels. (C) Processed time lapse images at specific timepoints, 93 min (time point 31), 126 min (time point 126); 192 min (time point 64), with combined red and green channels. Processing included flat field correction, background fluorescent subtraction and cross talk subtraction. Arrows included in images direct to specific locations where false signal is removed/reduced during processing. Single channel images for processed time-lapse image of SYBR Gold stained Nf phage (cyan) and *Bacillus subtilis* expressing mKate fluorescence (magenta) are found in Methods Video S3. Single channel images for processed time-lapse image of SYBR Gold stained Nf phage (cyan) and *Bacillus subtilis* expressing mKate fluorescence (magenta) are found in Figures S3.

Preparation 4: The expected outcome of the images post-processing is a corrected time-lapse showing bacterial cell growth and death following phage infection and host-cell lysis (Methods video S3) Removing both the background fluorescence and cross-talk enables better localization of specific SYBR Gold signal (Figures 4B and 4C, see also Figure S3). This enables more reliable interpretation of phage localization and infection dynamics across the time-lapse.

Methods Video S3. Time lapse for *Bacillus subtilis* cell lysis by NF phage *Bacillus subtilis* expressing mKate fluorescence combined with SYBR™ Gold-stained NF phage (1:1), red (magenta coloring) and green (cyan coloring) channel combined, after post processing.

## Limitations

### Definitions

**cross-talk** – fluorescence from one channel is being detected in another channel. Can be attributed to overlap in emission spectra, broad filter settings and strong signal intensity.

**auto-fluorescence**- the natural occurring fluorescence of objects.

In these experiments, the fluorescent signal emitted from the SYBR Gold stained phage particles is significantly weaker (<300 a.u.) than the bacterial mKate signal (>1000 a.u.). Stained phage signal is also weakened by phage mobility, which also makes it challenging to observe the phage because they keep moving out of focus. mKate itself is has an optimally excitation maximum at ∼588 nm, with a broad excitation spectrum that extends into lower wavelengths (around 442, depending the version of mKate). This results in small overlap with the 488 nm green channel laser. Because of the small excitation range overlap and difference in signal strength, it was observed that cross-talk from the red channel (mKate signal) appeared in the green channel. Auto-fluorescence from the bacteria in the green channel was ruled out by imagining the same bacteria strain lacking the fluorescent gene. While a small amount of auto-fluorescence was observed, the signal strength was much lower than the cross-talk fluorescence observed.

Adjusting brightness and contrast can visibly reduce background fluorescence; however, it does not correct cross-talk and may also reduce visibility of true signal from the stained phage. For accurate presentation and quantification of fluorescent images/time-lapse results, cross-talk and background fluorescence should be subtracted during image postprocessing. It should be noted that for the post processing details here, the corrections were aimed in reducing the background fluorescence and cross-talk as much as possible without affecting the strained phage signal. Hence, due to the weakness of the strained phage signal, the corrections were lenient. Detailed steps for removing background fluorescence and reducing cross-talk are provided in Preparation 4.

## Troubleshooting

### Problem 1

Agarose pad instability during time lapse imaging including movement, shrinkage, bubbles and condensation.

### Potential solution

Completely encasing the pad securely in the frame and cover slip is usually sufficient to prevent spaces which allow the pad to move around or for condensation to form. Manual practice of placing the cover slip helps reduce the chances of trapping gas under the cover slip to form bubbles. An additional solution to increase pad stability that was not explored during development is to increase the percentage of agarose from 1.5% to 2%. (Related to Preparation 3, step 2 k).

### Problem 2

High background fluorescence reduces the phage particle contrast and may result in false-positive fluorescent signals.

### Potential solution

High background fluorescence and false-positive fluorescent signals can be caused by residual unbound dye in the sample and the dye binding to an unintended target. When working with unfamiliar dye using these protocols additional preliminary steps are required to confirm what the level of unbound dye is low and that the dye is only bound to the intended target. This can usually be achieved using negative control samples and comparison samples, like demonstrated in Figure 2. (Related to Preparation 2, step 1 and step 2)**.**

### Problem 3

Phage has detectable fluorescence, but no host-cell death or lysis is observed in co-cultures.

### Potential solution

The binding method of the dye or location of a fluorescent tag could affect the phage or host in such a way that it prevents infection or phage replication. An example of this is a dye that binds to the receptors that are responsible for phage attachment. It is strongly recommended that when using an unknown dye or newly tagged biological sample to confirm that the phage are still capable of infection and replication. This can be achieved by performing a plaque assy. (Related to Preparation 2, step 2. v. critical).

### Problem 4

Black spots appearing on images during postprocessing cross-talk subtraction.

### Potential solution

Black spots or locations with large negative values that appear after cross talk removal usually occur when too much signal has been subtracted. This can be due to the R_scale_k image being incorrectly generated. (Related to Preparation 4, step 4. f).

### Problem 5

Cells fail to grow within the imaged field of view but are observed to be growing in the surrounding regions.

### Potential solution

This problem could indicate that the laser/s being used are causing cell distress and inhibiting their growth. Adjust the laser power and exposure time to identify the best combination for reducing growth inhibition while maintaining a reliable fluorescence observation. Reducing the laser power first to minimize light-induced stress then adjust the exposure time if the signal becomes too weak. Choosing multiple locations will increase the likelihood of selecting positions with healthier cells in case the sample preparation is also inducing stress. (Related to Preparation 3, step 3).

## Resource availability

### Lead contact

Further information and requests for resources and reagents should be directed to and will be fulfilled by the lead contact, Anna Dragoš (anna.dragos@bf.uni-lj.si).

### Technical contact

Technical questions on executing this protocol should be directed to and will be answered by the technical contact, Hannah R. Bonham (hannah.bonham@bf.uni-lj.si).

### Materials availability

This study did not generate new unique reagents.

### Data and code availability

The raw image data supporting the current study have been deposited at Zenodo: https://zenodo.org/records/18165781.

## Acknowledgments

We would like to deeply thank Prof. Marc Bramkamp and Dr. Helge Feddersen for their guidance during the first experiments with agarose pads and for their feedback on this manuscript. H.R.B., V.G., V.A.F., and A.D. were funded by the European Union (ERC, PHAGECONTROL, 101041421). Views and opinions expressed are, however, those of the authors only and do not necessarily reflect those of the European Union or the European Research Council Executive Agency. Neither the European Union nor the granting authority can be held responsible for them. H.R.B. was additionally funded by the European Union (ERA Fellowship, Phage_Attach, 101244217). A.D., V.A.F., V.G., and I.D. were also supported by the Slovenian Agency for Research and Innovation (grant no. P4-0116).

## Author contributions

H.R.B., V.G., and V.A.F. performed a series of experiments on the described phage-bacteria model system that led to the final protocol. H.R.B. performed specific experiments described in the manuscript and was the most invested in protocol optimization. All authors contributed to different steps of protocol optimization; I.D. provided further feedback on optimal oxygenation, A.D. supervised the project, H.R.B. wrote the manuscript, and all authors contributed to final version.

## Declaration of interests

The authors declare no conflict of interest.
