# Supplementary material for: Optimized protocol for live imaging of Bacillus subtilis phage infection at a single-cell resolution
Source: STAR Protoc. 2026 Aug 26;7(3):104805. doi: 10.1016/j.xpro.2026.104805 (PMC13543884; doi:10.1016/j.xpro.2026.104805)
Supplement: Supplementary file 1 [file mmc1.pdf]

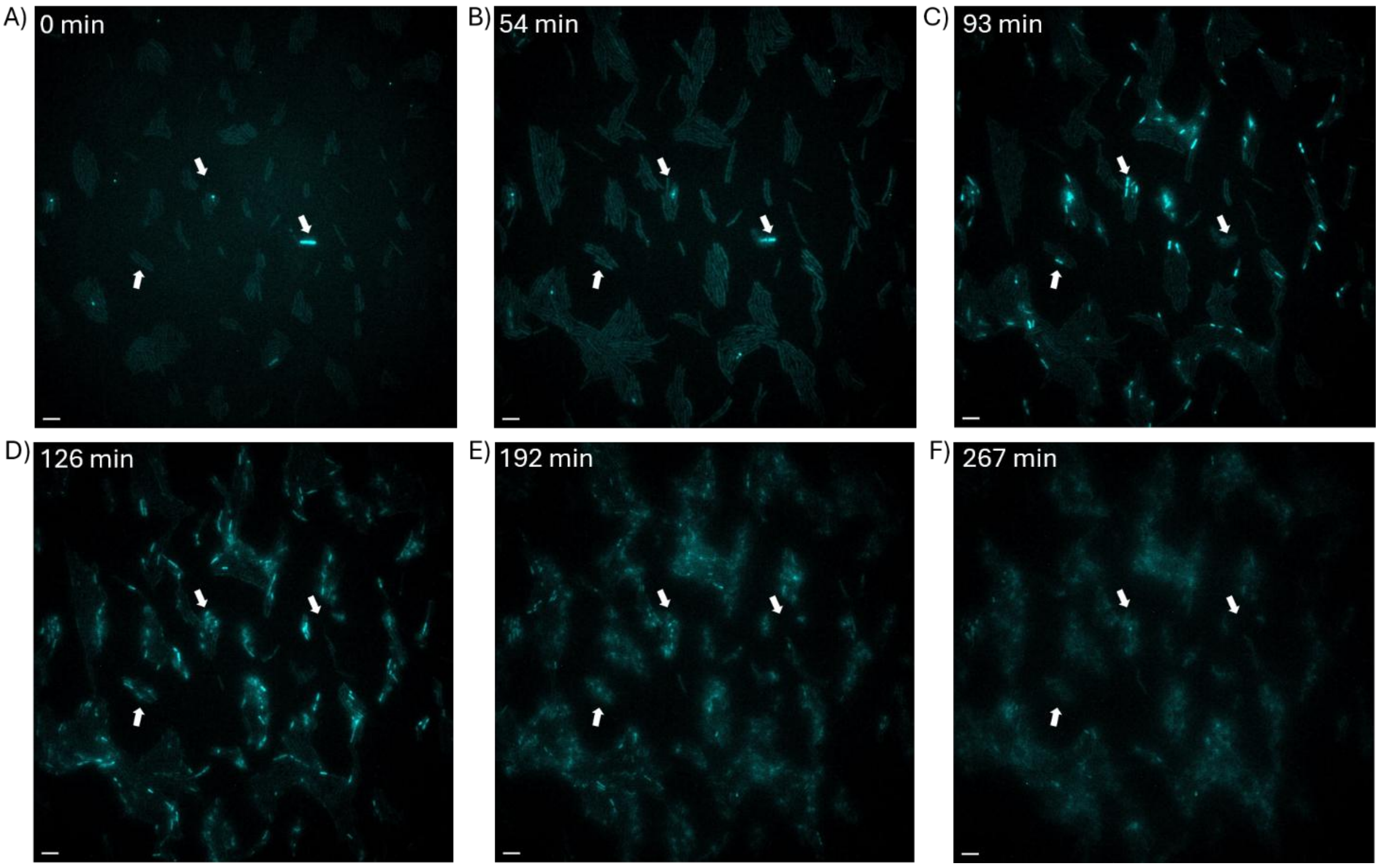

Figure S1: Green channel images (cyan) of time-lapse microscopy imaging of *Bacillus subtilis* expressing mKate fluorescence (magenta) combined with SYBR™ Gold stained Nf phage (cyan), related to Figure 3. All scale bars (top left corner) present in microscope images are 5 µm. Arrows included in images direct to specific bacterial cells. Green (488 excitation wavelength) fluorescence channel, A) time point 0 minutes ; B) time point 18 (54 minutes); C) time point 31 (93 minutes); D) time point 42 (126 minutes); E) time point 64 (192 minutes); F) time point 89 (267 minutes). Images are described as raw images since they still contain background fluorescent signal and cross-talk.

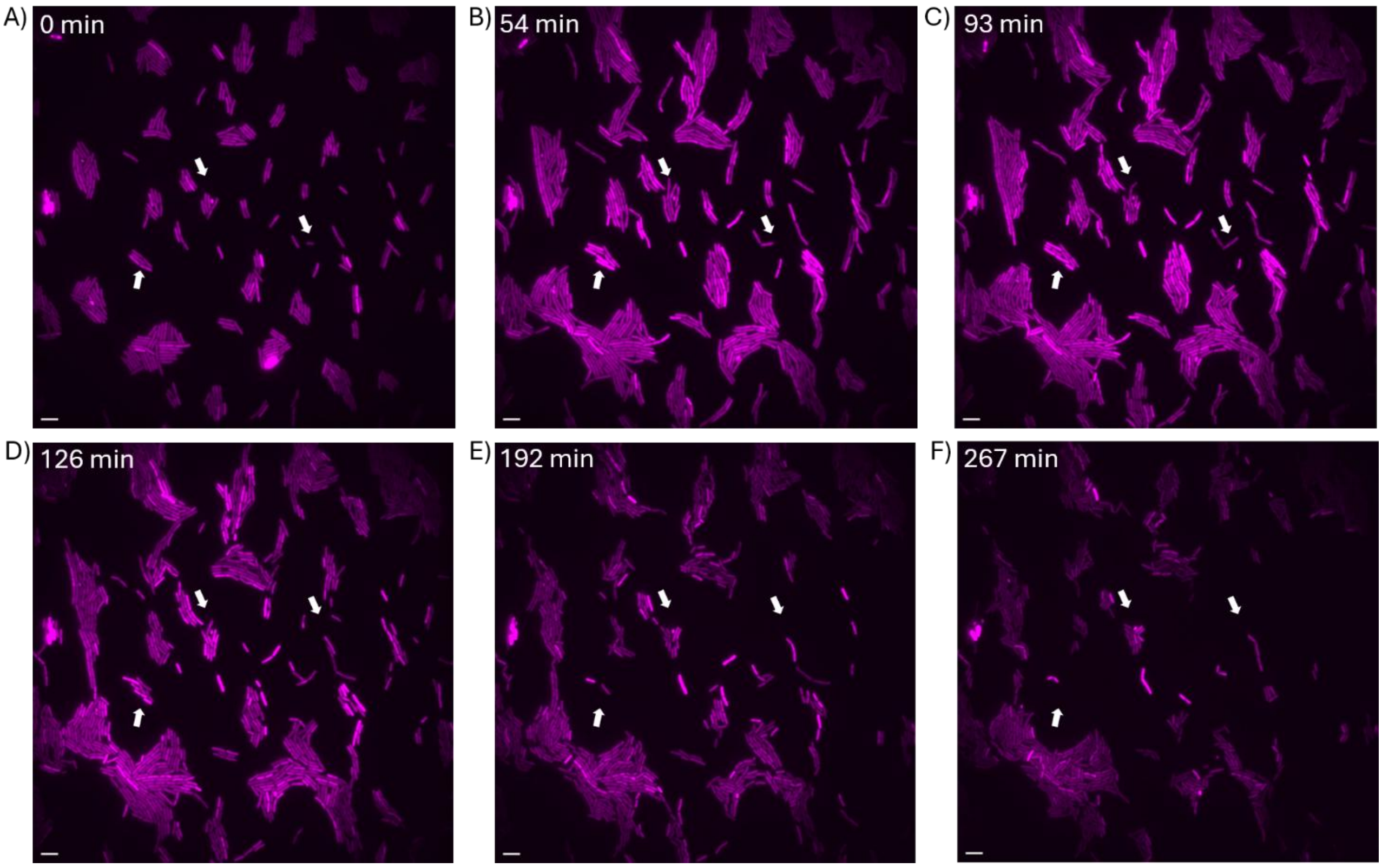

Figure S2: **Red channel images (magenta) of time-lapse microscopy imaging of *Bacillus subtilis* expressing mKate fluorescence (magenta) combined with SYBR™ Gold stained Nf phage (cyan), related to Figure 3.** All scale bars (top left corner) present in microscope images are 5 µm. Arrows included in images direct to specific bacterial cells. Red (561 excitation wavelength) fluorescence channel, A) time point 0 minutes ; B) time point 18 (54 minutes); C) time point 31 (93 minutes); D) time point 42 (126 minutes); E) time point 64 (192 minutes); F) time point 89 (267 minutes). Images are described as raw images since they still contain background fluorescent signal and cross-talk.

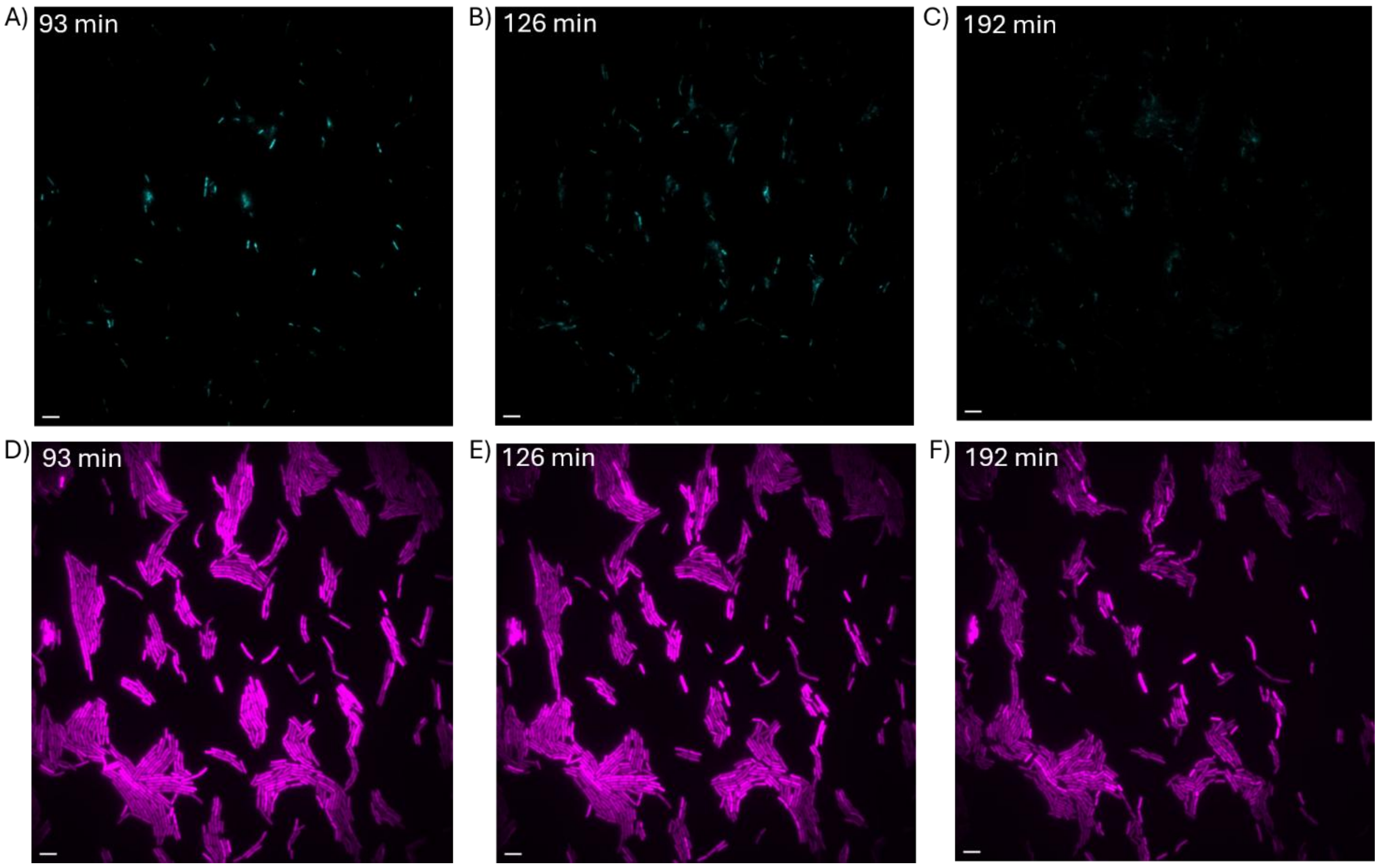

Figure S3: **Separated channels for processed time-lapse image of *Bacillus subtilis* expressing mKate fluorescence (magenta) combined with SYBR Gold stained Nf phage (cyan), related to Figure 4**. All scale bars (top left corner) present in microscope images are 5 μm. All images include post processing for flat field correction, background fluorescent subtraction and cross talk subtraction. A), B), and C) contain specific timepoints, 93 minutes (time point 31), 126 minutes (time point 126); 192 minutes (time point 64) for the green channel images (cyan). D), E) and F) contain specific timepoints, 93 minutes (time point 31), 126 minutes (time point 126); 192 minutes (time point 64) for the red channel images (magenta).
